# Blue and Yellow Laccases from *Alternaria* sp. Strain HU: Characterization and Immobilization on Magnetic Nanoparticles

**DOI:** 10.3390/jof10080559

**Published:** 2024-08-08

**Authors:** Ingrida Radveikienė, Regina Vidžiūnaitė, Rolandas Meškys, Vida Časaitė

**Affiliations:** Life Sciences Center, Institute of Biochemistry, Vilnius University, Sauletekio Av. 7, 10257 Vilnius, Lithuaniarolandas.meskys@bchi.vu.lt (R.M.);

**Keywords:** laccase, yellow laccase, *Alternaria* sp., isoenzymes, flavonoids, catalytic properties

## Abstract

Laccases are important and valuable enzymes with a great potential for biotechnological applications. In this study, two novel laccases, LacHU1 and LacHU2, from *Alternaria* sp. HU have been purified and characterized. The molecular mass of each isoenzyme was ~66 kDa. LacHU1 laccases was yellow and had no typical blue oxidase spectra and LacHU2 had a blue color and characteristic absorption spectra. The catalytic efficiency of LacHU1 for most substrates was higher than that of LacHU2 laccase. Both isoenzymes effectively oxidize flavonoids. *Alternaria* sp. laccases were successfully immobilized on magnetic nanoparticles. The thermostability of immobilized laccases increased and optimal pH shifted to more alkaline compared to the free laccases. Potential applications of laccases from *Alternaria* sp. HU are in the oxidation of flavonoids in cotton or in water treatment processes.

## 1. Introduction

With the need for a more sustainable way of life, biocatalysis is at the forefront as an environmentally conscious approach. From environmental remediation to industrial biotechnology and health care, enzymes are recognized as versatile tools. Among them, laccase stands out as one of the most adaptable catalysts due to its exceptional properties. Laccases (EC 1.10.3.2, *p*-diphenol: dioxygen oxidoreductase) are copper-containing enzymes that catalyze the oxidation of a wide range of phenolic and non-phenolic compounds using molecular oxygen as an electron acceptor [1]. In recent decades, laccases excel in the oxidation of flavonoids, valorization of lignocellulosic compounds, bleaching of wood pulp and removal of undesirable phenolics that cause browning, haze and turbidity in fruit juices and wine [2,3,4]. In addition, laccases have shown efficacy in diverse applications such as decolorizing textiles, degrading olefinic plastic waste, catalyzing organic synthesis reactions and biosensor development [5,6,7,8,9,10,11,12]. Despite the discovery of many laccases synthesized by different organisms, the search for new laccases is driven by the need to find enzymes with improved properties and tailored functionality. Fungi are excellent producers of new laccases with the required properties for industrial applications [13,14,15], and ascomycetes are known to be able to synthesize several laccases isoenzymes with different catalytic and physicochemical properties. The study of laccase synthesized by fungi has led to the discovery of many useful and unique enzymes, such as active at neutral pH [16], halophilic [17] and thermostable [18]. Thanks to the discovery of enzymes and their unique properties, their use in a wide range of biotechnology applications is now even more widespread. The isoenzymes present in a given organism add further diversity. Studies show that fungi can have up to eighteen different laccase genes in their genome [19]. Additionally, the proteomic analysis shows that not all genes are synthesized, indicating a different induction of laccase [20,21].

The number and composition of laccase isoforms synthesized by fungi are highly dependent on the fermentation conditions. Previous studies have shown that some laccase isoenzymes are constitutively expressed and some are inducible. Metal ions such as copper or manganese, and various aromatic compounds can induce one or multiple laccase isoenzymes [22,23,24]. In addition, some fungi secrete not only traditional blue laccases but also less common yellow ones [25]. Yellow laccases can oxidize non-phenolic lignin compounds, which cannot be oxidized by the blue laccase alone [26]. This provides an advantage in the application of such laccases in the industry as it expands the range of substrates and does not require additional mediators.

Immobilization allows the biocatalyst to be reused or the performance of the enzyme to be optimized, contributing to sustainable and cost-efficient processes. Immobilization may reduce the sensitivity of the enzyme to environmental conditions. Nanoparticles exhibit characteristics that are ideal for immobilization, such as high surface to volume ratio, effective enzyme loading and diffusion minimization [27,28]. Iron oxide magnetic nanoparticles (Fe_3_O_4_) have received considerable attention due to their easy synthesis, strong magnetic properties, low toxicity, and low cost [29].

In our study, we isolated *Alternaria* sp. fungus from soil and investigated two laccases synthesized under different growing conditions. Among these, we identified a yellow laccase capable of oxidizing a broader spectrum of compounds compared to typical laccases. Additionally, we characterized another inducible laccase exhibiting excellent catalytic activity even in environments with high salt concentrations. This property is particularly useful in applications such as water treatment, where salt levels are often elevated. Both laccase isoenzymes LacHU1 and LacHU2 from *Alternaria* sp. HU were characterized and immobilized on magnetic nanoparticles.

## 2. Materials and Methods

### 2.1. Materials

2,2’-azino-*bis*(3-ethylbenzothiazoline-6-sulfonic acid) diammonium salt (ABTS), promazine hydrochloride, syringaldazine, 1-naphthol, kaempferol, myricetin, quercetin, fisetin, gallic, syringic, chlorogenic acids, L-3,4-dihydroxyphenylalanine (L-dopa), metal salts, and hydrogen peroxide were purchased from Sigma-Aldrich, Buchs, Switzerland. 2,6-dimethoxyphenol was obtained from Alfa Aesar, Kandel, Germany. Ferulic and caffeic acids were obtained from Fluka, Steinheim, Switzerland. *N,N’*-dimethylamine-4-(4-morpholine)benzene and 2-(10*H*-phenoxazin-10-yl)ethanol were prepared as described [30]. Catechol, hydroquinone, *p*-phenylenediamine, potassium hexacyanoferrate (II) were obtained from Reachim, Moscow, Russia.

### 2.2. Media and Culture Conditions

Fungal strain HU was isolated from soil on the malt extract agar medium ME (2% of malt extract, 2% of agar and 0.4% of yeast extract) supplemented with 1 mM ABTS. The fungus was transferred to the fresh ME and stored at 20 °C monthly. The pre-culture medium consisted of (g L^−1^): malt extract—3.5, yeast extract—2.5, MgSO_4_—0.5, glucose—8.0, KH_2_PO_4_—2.0, pH 6.0. The LacHU1 medium consisted of (g L^−1^): malt extract—3.5, yeast extract—2.5, MgSO_4_—0.5, glucose—1.0, (NH_4_)_2_SO_4_—2.0, pH 5.0. The LacHU2 medium consisted of (g L^−1^): malt extract—3.5, yeast extract—2.5, MgSO_4_—0.5, glucose—1.0, KH_2_PO_4_—20.0, pH 4.0. The media were sterilized by autoclaving at 121 °C for 20 min.

For laccase induction, autoclaved CuSO_4_ (1.0 mM in the LacHU1 medium and 1.5 mM in the LacHU2 medium) and filter sterilized 3,4-dimethylaniline (1 mM in the LacHU2 medium) were added to the sterile media prior to cultivation.

Mycelia taken from agar plates were grown in a pre-culture medium for 4 days. The Erlenmeyer flasks with 200 mL of LacHU1 and LacHU2 media were inoculated with 8 mL of liquid fungal pre-culture suspension and cultivated for 4 days (LacHU1) or 5 days (LacHU2). The cultivation was carried out on a rotary shaker at 180 rpm and 30 °C.

For the evaluation of the inducer effect, the LacHU1 and LacHU2 media were supplemented with 1 mM of benzotriazole, *p*-aminobenzoic acid, *N,N*-diethylaniline, 2,3-dimethylaniline, *N*-ethyl-5-hydroxy-2-methylaniline, aniline, *N,N-*dimethylaniline, 2,5-dimethylaniline, *p*-dimethylbenzaldehyde, 3-dimethylamino phenol, 4-nitroaniline, or 3,4-dimethylaniline. All inducers were sterilized by passing through a 0.22 µm polyethersulfone syringe filters (Whatman, Maidstone, UK) before addition to the sterilized medium. The laccase activity was measured after 4 days of cultivation.

### 2.3. Identification of Fungus

Fungal strain HU was identified by the 18S rRNA gene sequencing as described previously [31]. The nucleotide sequence was deposited in the GenBank under the accession number MZ350812. The 18S region sequence was then used to carry out BLAST on the nucleotide database of NCBI (http://www.ncbi.nlm.nih.gov (accessed on 11 June 2021)). Based on maximum identity score, 31 nucleotide sequences were selected for preparing the phylogenetic tree. The evolutionary history was inferred by using the Maximum Likelihood method based on the Tamura–Nei model [32]. Initial tree for the heuristic search were obtained automatically by applying the Neighbor-Joining and BioNJ algorithms to a matrix of pairwise distances estimated using the Maximum Composite Likelihood (MCL) approach, and then selecting the topology with superior log likelihood value. The tree was drawn to scale, with branch lengths measured in the number of substitutions per site. There were a total of 2381 positions in the final dataset.

For laccase gene identification, the laccase proteins were subjected to the MS/MS analysis as described previously [31]. The identified peptide sequences were used in a blast search (https://blast.ncbi.nlm.nih.gov/Blast.cgi (accessed on 12 April 2022)) and the closest homologous sequences were used to select motifs at the ends of the genes to create primers. Specifically, the primers HU2YF (5′-ATGTTGGTTCYTTCATTAGC-3′) and HU1YR (5′-TYAGATACCAGTCAGTCAGTCTG-3′) were generated for LacHU1 laccase, whereas the primers HU2BF (5′-ATGCTTCGCKGTATCTTC-3′) and HU2BR (5′-CTAWACACCGGGAGTCATCCTG-3′) were generated for lacHU2 laccase. The laccase genes were amplified from chromosomal DNA and the resulting fragments sequenced. LacHU1 and LacHU2 sequences were submitted to the GenBank under the accession numbers PP756381 and PP756382.

For phylogenetic analysis of laccase, the annotated laccase amino acid sequences from the genomes of *Alternaria* spp. (*A. aborescens* ASM415483v1, *A. gaisen* ASM415602v2, *A. tenuissima* ASM415603v1, *A. burnsii* ASM1303605v1, *A. alternata*, GCA_014751505.1, GCA_001642055.1, *Alternaria* sp. MG1 GCA_003574525.1) were used. The evolutionary history was inferred using the Minimum Evolution method [33]. The evolutionary distances were computed using the number of differences method [34] and are in the units of the number of amino acid differences per sequence. The ME tree was searched using the Close-Neighbor-Interchange (CNI) algorithm [34] at a search level of 1. The Neighbor-Joining algorithm [35] was used to generate the initial tree. The analysis involved 26 amino acid sequences. Evolutionary analysis was conducted in MEGA7 [36]. Protein molecular weight were calculated from amino acid sequence by the Sequence Manipulation Suite (https://www.bioinformatics.org/sms/index.html (accessed on 15 June 2024)), the introns were predicted by the Augustus software available at https://bioinf.uni-greifswald.de/bioinf/ (accessed on 15 June 2024). Signal peptides were predicted by SignalP 5.0 [37]. Glycosylation was predicted by NetNGlyc—1.0 [38].

### 2.4. Purification of Laccases

The grown culture was collected and centrifuged at 1500× *g.* The cleared culture liquid was concentrated by tangent ultrafiltration with 30 kDa cut-off filter (Millipore, Burlington, MA, USA) and up to 2.2 M of (NH_4_)_2_SO_4_ was added to remove polysaccharides. The precipitates after 24 h of incubation at 4 °C were removed by centrifugation (4000× *g*) and pure supernatant was applied to a PHE-Sepharose FF (GE Healthcare, Chicago, IL, USA) column, equilibrated with 5 mM sodium citrate with 2.2 M (NH_4_)_2_SO_4_ buffer, pH 5.5. Retained protein was eluted by linear gradient of 2.2–0 M (NH_4_)_2_SO_4_ in sodium citrate buffer. Fractions with laccase activity were collected, concentrated, and dialyzed against the 5.0 mM sodium citrate buffer, pH 5.5 and applied on the Source Q15 column (16 mm × 25 mm, GE Healthcare, Chicago, IL, USA). LacHU1 and LacHU2 were eluted at 25 mM and 400 mM of (NH_4_)_2_SO_4_, respectively, by applying linear gradient of (NH_4_)_2_SO_4_ (0–0.5 M) in sodium citrate buffer, pH 5.5. Fractions with laccase activity were collected, concentrated and dialyzed against 5 mM sodium citrate buffer, pH 5.5. LacHU1 was additionally purified on Source 15PHE column (GE Healthcare, Chicago, IL, USA). LacHU1 was eluted with linear gradient of 1.5–0 M (NH_4_)_2_SO_4_, fractions with activity were collected, concentrated and dialyzed against 5.0 mM sodium citrate buffer, pH 5.5. Both purified enzymes were stored at –20 °C.

### 2.5. Characterization of Laccases

The UV–VIS absorption spectra of the purified laccases were determined as described previously [31]. SDS-PAGE was performed according to the method of Laemmli [39] in 14% polyacrylamide gels by using the molecular mass standard (Pierce™ Unstained Protein MW Marker, ThermoFisher Scientific, Vilnius, Lithuania). Protein concentration was determined by the method of Lowry using bovine serum albumin as a standard [40]. MS/MS analysis was performed as described previously [31]. The molecular mass was estimated by SDS-PAGE.

### 2.6. Enzyme Activity Assay

The activity of laccase was determined with ABTS as substrate as described previously [31] in 50 mM sodium acetate buffer, pH 5.5. These measurements were performed to determine activity in the medium, during purification steps, after stability and immobilization experiments.

The oxidation of the selected substrates, tyrosinase and peroxidase activity was estimated as described in [31].

The activity of laccase at different pH values was determined in a pH range of 2.5–8.5 using a universal 60 mM Britton–Robinson buffer [41].

Optimal temperature was determined by measuring the activity at 25–70 °C. For the determination of the thermal stability, laccases were incubated at 25–60 °C for 10 min and the residual activity was measured with ABTS as a substrate. 

The effect of metal salts on laccases stability was determined after 10 min of incubation in 50 mM sodium acetate buffer solution (at pH 5.0 for LacHU1 and pH 3.0 for LacHU2) supplemented with 1.0 mM of different metal salts at 25 °C. The effect of metal salts on laccases activity was determined in the presence of 1 or 10 mM of CaCl_2_, CoCl_2_, CuCl_2_, MnCl_2_, NiCl_2_, CdI, Li_2_SO_4_, ZnSO_4_, and Pb(CH_3_COO)_2_ using ABTS assay.

The Michaelis–Menten equation was used to analyze the dependence of the initial reaction rate on the substrate concentration and to determine the apparent kinetic parameters V_max_ and K_M_ of the reactions. The catalytic constant (k_cat_) was calculated as a ratio of V_max_ and the total enzyme concentration. The bimolecular enzyme and substrate reactivity constant (k_cat_/K_M_) were calculated as a ratio of the catalytic constant (k_cat_) and K_M_.

### 2.7. Immobilization of Laccases on the Fe_3_O_4_ Nanoparticles

The synthesis of nanoparticles and immobilization of laccases were performed as described [42]. Briefly, 10 mg/mL of sonicated Fe_3_O_4_ nanoparticles were mixed with different concentrations of an enzyme (4.6–17.5 nM) in a volume ratio of 1:1 and were incubated from 10 min to 120 min at room temperature. Then, 20 μL of the nanoparticle/laccase mixture was separated by the magnet, 20 µL of fresh buffer (50 mM sodium acetate buffer, pH 5.5) was added to the nanoparticle fraction and both fractions (supernatant and nanoparticles) were used for activity measurement in 2 mL of reaction solution using ABTS as a substrate. The highest concentration of enzyme (~9 nM, all the enzyme was immobilized on the nanoparticles and no enzymatic activity remained in the supernatant) was used for the reusability and kinetic assays. To calculate the concentration of active enzyme on the nanoparticles (C_MNPs_), we measured the reaction rate of free enzyme (V_free_) using a known concentration of enzyme solution (C_free_) and used the same concentration of enzyme for immobilization. After measuring the reaction rate of immobilized enzyme (V_MNPs_), we calculated C_MNPs_ from the rate ratio:

$$C_{MNPs}=\left(C_{free}\cdot V_{MNPs}\right)/V_{free}$$

The immobilization efficiency (IE) was calculated according to the following formula:

$$IE(\%)=P/P_{o}\cdot 100\%$$

wherein *P* was the activity on ABTS of the immobilized enzyme and *P*_o_ was the activity of initial enzyme sample. The effectiveness factor was calculated according to the following formula:

$$Effectiveness\;factor=V_{\max(immobilized\;enzyme)}/V_{\max(free\;enzyme)}$$

For the reusability experiment, the immobilized enzyme was collected from the cuvette with the magnet, suspended in 20 µL of fresh sodium acetate buffer solution and used for the re-measurements up to 10 reaction cycles.

## 3. Results

### 3.1. The Biosynthesis, Identification and Purification of Laccases 

**Figure 1:**
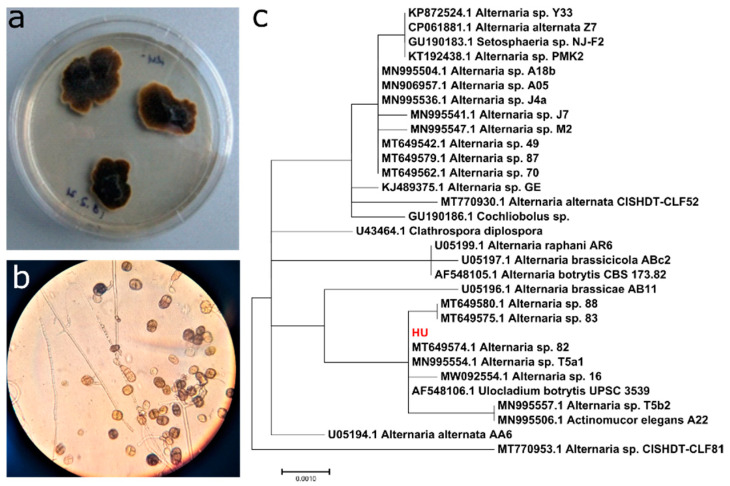
*Alternaria* sp. HU strain. (**a**) The colonies on the ME medium. (**b**) Microscopic view of hyphae and conidia. (**c**) Phylogenetic tree constructed from the alignment of the closes related 18S rRNA encoding sequences (HU indicated in red), using the Maximum Likelihood method; the tree was generated using MEGA7.

In the search for new laccases, a fungal strain HU that oxidizes ABTS on solid media (ME) was isolated from soil. The texture of the HU strain colonies was velvety and the color varied from brown to black. In the microscopic studies, septated conidia were visible. Analysis of the small subunit ribosomal RNA gene sequence (GenBank accession number MZ350812) indicates the closest match (99–100% similarity) with over 90% sequences of different *Alternaria* species. The phylogenetic analysis confirm that the strain HU belongs to the genus *Alternaria* (Figure 1).

**Figure 2:**
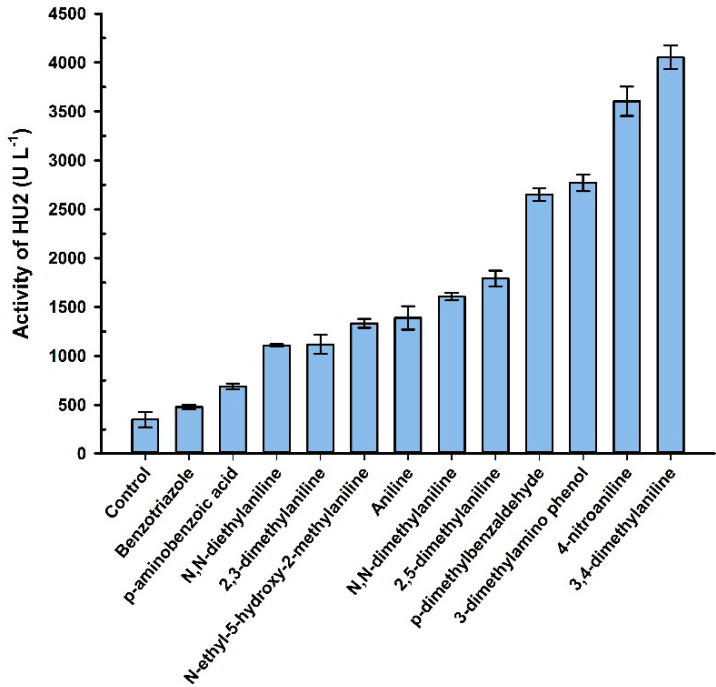
The effect of inducers on LacHU2 laccase biosynthesis. The LacHU2 medium containing 1.5 mM CuSO_4_ (50 mL) was supplemented with 1 mM of benzotriazole, *p*-aminobenzoic acid, *N,N*-diethylaniline, 2,3-dimethylaniline, *N*-ethyl-5-hydroxy-2-methylaniline, aniline, *N,N-*dimethylaniline, 2,5-dimethylaniline, *p*-dimethylbenzaldehyde, 3-dimethylamino phenol, 4-nitroaniline or 3,4-dimethylaniline and cultivated as described in Materials and Methods (N = 3).

An analysis of liquid culture (grown in the LacHU1 medium supplemented with 1 mM CuSO_4_) indicated that *Alternaria* sp. HU secreted laccases into the medium. During the purification of enzyme, two active fractions separated with a different retention on the anionic exchanger SourceQ (Table 1). As a result, we created two different media to find optimal growing conditions for the individual laccases. Laccases were assigned as LacHU1 and LacHU2. It turned out, that the pH and salt concentration of the medium were the most important factors for the production of enzyme: the high concentration of phosphates stimulated the synthesis of LacHU2 and completely inhibited the synthesis of LacHU1; the lower pH (pH 4) was more preferable for LacHU1 and the higher pH (pH 5) for LacHU2. Production of both laccases was significantly stimulated by 1.0–1.5 mM copper, with a 72- and 22-fold increase in activity in LacHU1 and LacHU2 media, respectively. Moreover, some aromatic compounds stimulated the synthesis of LacHU2 and increased its activity up to ten-folds (Figure 2). The highest activity of laccase was observed when 1 mM of 3,4-dimethylaniline or 4-nitroaniline were added to the medium. However, the inducers studied did not increase the synthesis of LacHU1.

Medium optimization allowed achieving 600 U L^−1^ of LacHU1 and 8000 U L^−1^ of LacHU2 laccase. After purification from one liter of culture, we obtained 1.0 mg of LacHU1 (with a specific activity of 142 U mg^−1^) and 3.3 mg of LacHU2 (with a specific activity of 1630 U mg^−1^) laccase (Table 1).

### 3.2. Characterization of Laccases 

**Figure 3:**
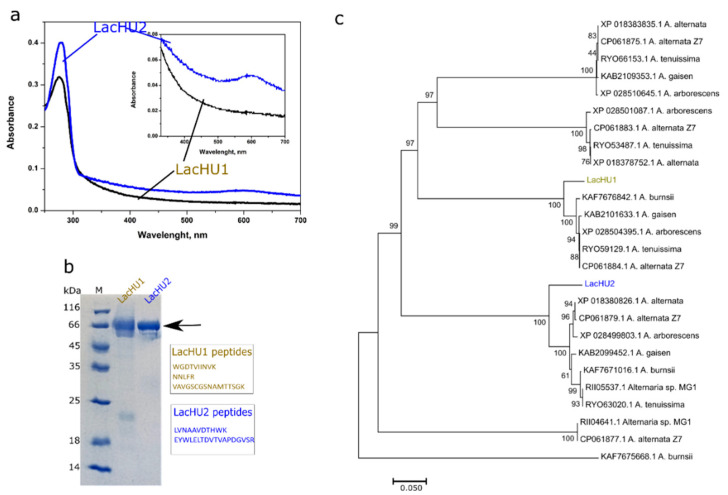
Analysis of LacHU1 and LacHU2 laccases. (**a**) The UV–VIS spectrum of the purified laccases. (**b**) SDS-PAGE analysis of the purified laccases, and the identified peptides by the MS/MS analysis; M—molecular mass marker. (**c**) Phylogenetic tree of *Alternaria* sp. laccases.

The purified LacHU1 sample was light yellow and had no peak at the 610 nm in the UV–VIS spectrum. A slight shoulder at 330 nm in this spectrum suggests the presence of a T3 copper site (Figure 3a). In contrasts, the LacHU2 laccase exhibited a typical blue color of copper oxidase and an absorbance peak at 610 nm (Figure 3a). The molecular mass of both enzymes determined by SDS-PAGE was ~66 kDa. The laccase bands are smeared in SDS-PAGE, presumably because proteins are glycosylated. An MS/MS analysis of both laccases revealed several tryptic peptides. A BLAST analysis of the NCBI protein database using the obtained peptide sequences allowed the conclusion that LacHU2 and LacHU1 proteins were different *Alternaria* laccases.

We designed primers based on homologous sequences, amplified both laccases by PCR and determined their sequences. The sequences were compared with known *Alternaria* sp. laccases. According to the phylogenetic analysis LacHU1 and LacHU2 (Figure 3b) clustered with laccases from different branches (Figure 3c). LacHU1 and LacHU2 were 37.7% identical at the amino acid level and 51.3% identical at the DNA level. 

Sequence analysis also revealed elements common to many laccases: both enzymes have signal peptides of 18 amino acids in length, five possible N-glycosylation sites each. The genes encoding laccases contain introns—one in *lacHU1*, and two in *lacHU2*. The molecular weight calculated from the sequence (without the signal peptides) was 63.5 kDa for LacHU1 and 59.5 kDa for LacHU2, which is lower than that determined by SDS-PAGE (around 66 kDa), probably due to glycosylation of the enzymes.

### 3.3. Catalytic Properties of the LacHU1 and LacHU2 

In order to characterize the LacHU1 and LacHU2 isoenzymes, the oxidation of various organic substrates was analyzed. Both enzymes were active towards phenolic, e.g., quercetin, myricetin, kaempferol, 2,6-DMP, hydroquinone, catechol, as well as non-phenolic heterocyclic compounds, e.g., ABTS, syringaldazine, promazine hydrochloride; and inorganic compound potassium hexacyanoferrate (II) (see Supplementary Figure S1). The highest K_M_ values differed: for LacHU1 laccase, the highest K_M_ was for promazine and pyrocatechol (Table 2), and for LacHU2 it was for potassium hexacyanoferrate (II) and catechol, indicating a low affinity for these substrates. The catalytic efficiency (k_cat_/K_M_) and affinity of LacHU1 laccase were significantly higher than those of LacHU2 laccase for all substrates studied.

LacHU1 had a higher catalytic efficiency for ABTS and 2,6-DMP than non-blue laccase from *Methylobacterium extorquens, Pleurotus ostreatus, Phellinus ribis, Bacillus* sp. MSK-01, *Myrothecium verrucaria* NF-05 and *Trametes hirsute.* In addition, LacHU1 had a higher k_cat_ with ABTS and 2,6-DMP than the non-blue laccase from *Methylobacterium extorquens* [43]. Neither peroxidase nor tyrosinase activity was detected for both laccases. LacHU1 did not oxidize veratryl alcohol. LacHU1 was active at lower pH (with syringaldazine, 1-naphthol, ferulic acid, and myricetin), whereas LacHU2 was active at higher pH values (Supplementary Figure S2).

### 3.4. Influence of Metal Salts on the Activity of Laccase

**Figure 4:**
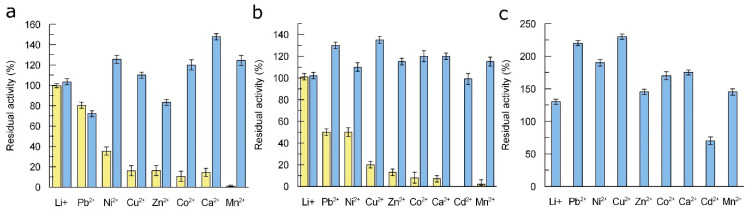
The effect of metal salts on stability and activity of LacHU1 and LacHU2. (**a**) Residual activity of LacHU1 and LacHU2 incubated with metal salts at room temperature for 10 min. (**b**) The activity of LacHU1 and LacHU2 in the presence of 1 mM of metal salts. (**c**) The activity of LacHU2 in the presence of 10 mM of metal salts. The 100% represents the activity without salts; N = 3. LacHU1—yellow bars and LacHU2—blue bars.

Since high salt concentrations were required for the synthesis of LacHU2, we decided to find out how both enzymes tolerate ions of various metals. The effect of metal salts on the stability of LacHU1 and LacHU2 was determined by pre-incubating the enzyme in the presence of 1 mM of Li^+^, Pb^2+^, Ni^2+^, Zn^2+^, Cu^2+^, Ca^2+^, Co^2+^ and Mn^2+^ salts (Figure 4a). Different buffer pH values were used for LacHU1 and LacHU2, due to their different pH stability (see Supplementary Figure S3). LacHU2 was found to be more resistant to treatment than LacHU1. The activity of laccases was also measured in the presence of 1 mM or 10 mM of metal salts (Figure 4b,c). Again, the activity of LacHU1 was more affected than the activity of LacHU2.

LacHU2 activity in the presence of Mn^2+^ salt was significantly enhanced, as observed for the yellow laccase from the white-rot fungus *Coriolopsis gallica* NCULAC F1 (CGLac) [25]. Similar to LacHU2, the activity of CGLac was positively influenced by the presence of other metal ions, including Ca^2+^, Zn^2+^, Cu^2+^ and Pb^2+^. 

### 3.5. Immobilization of Laccases

**Figure 5:**
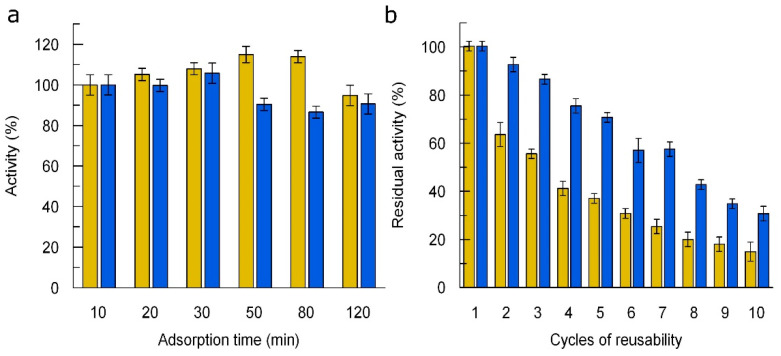
The effect of adsorption time (**a**) and reaction cycles (**b**) on the activity of immobilized LacHU1 and LacHU2 laccases. ABTS was used as substrate in 50 mM sodium acetate buffer solution, pH 5.5. LacHU1—yellow bars and LacHU2—blue bars (N = 3).

For multiple use, we immobilized the laccases on freshly synthesized magnetic nanoparticles by adsorption and evaluated three factors: enzyme concentration, adsorption time, and cycles of reusability. First, we explored the immobilization efficiency (IE) at different laccase concentrations. We determined that for both laccases, immobilization increased with increasing initial enzyme concentration up to 9 nM and started to decrease with increasing concentration above 9 nM. In addition, upon reaching this concentration, activity appeared in the nanoparticle supernatant, indicating that not all of the laccase was adsorbed (Supplementary Figure S4). By studying the effect of adsorption time on efficiency, we found that the activity of LacHU1 increased slightly up to 50 min and then gradually decreased. 30 min was the optimal reaction time for immobilization of LacHU2 (Figure 5a). As the activity of immobilized did not change much within 10–30 min, the immobilization time was set at 10 min. Under optimal conditions, the IE was 32% and 41% for LacHU1 and LacHU2, respectively.

The efficiency of reusability of the immobilized LacHU1 and LacHU2 was investigated over ten cycles of incubation with ABTS (Figure 5b). The activities of both laccases gradually decreased. After 10 cycles of reuse, LacHU1 and LacHU2 retained 18% and 38% of the initial activity, respectively.

**Figure 6:**
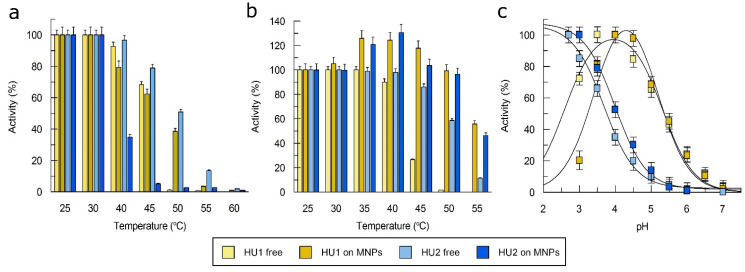
Comparison of the properties of free and immobilized HU laccases on magnetic nanoparticles (MNPs). (**a**) The effect of temperature on the activity of LacHU1 and LacHU2 laccases. (**b**) The activity of LacHU1 and LacHU2 laccases after 10 min incubation at various temperature. (**c**) The effect of pH on the activity of LacHU1 and LacHU2 laccases, using ABTS as substrate (N = 3).

To evaluate the effect of immobilization on the properties of LacHU1 and LacHU2, we compared the optimal temperature, optimal pH, thermostability, and kinetic constants of free and immobilized enzymes. The optimal temperature (25–30 °C) was identical for both laccases, but LacHU2 was more active than LacHU1 at elevated temperatures (Figure 6a). Both immobilized laccases were less active at high temperatures. When activity was evaluated after 10 min of incubation at different temperature, it was found that free LacHU2 was more thermostable than LacHU1, and immobilization provided thermostability to both laccases (Figure 6b).

A study of the effect of pH on activity showed that the optimal pH of both immobilized enzymes shifted to more alkaline compared to the free enzyme (Figure 6c).

The catalytic constants were determined for immobilized LacHU1 and LacHU2 and compared to the catalytic constant of free proteins (Table 3). The relative low K_M_ value of LacHU1 (compared to LacHU2) indicates its higher affinity for the ABTS. The K_M_ constant of immobilized and free LacHU1 were almost the same, although the catalytic efficiency (k_cat_/K_M_) of free LacHU1 laccase was significantly higher than that of the immobilized one. A possible explanation is that although the active site itself is unaffected, access to the immobilized LacHU1 active site is blocked. Similar results were obtained with the non-blue laccase Melac13220 [44]. The immobilized laccase had a higher K_M_ value than the free laccase, possibly due to the structural modification of the enzyme during the immobilization process or the loss of flexibility, which is important for optimal substrate binding. The catalytic efficiencies and K_M_ values of immobilized and free LacHU2 were almost identical. The immobilization effectiveness factor of LacHU1 was lower than that of LacHU2, specifically 0.27 and 0.5.

## 4. Discussion

In this study, we aimed to characterize the laccase isoenzymes, LacHU1 and LacHU2, from the strain *Alternaria* sp. HU. Many previous studies have reported the production of more than one laccase from the same fungal strain, usually encoded by different genes [22,24,31,45,46]. Between two and six putative laccase genes have been found in different genomes of *Alternaria* spp. The identified peptides of the LacHU1 and LacHU2 proteins were 100% identical to the amino acid sequences of two different laccase proteins from *Alternaria* sp. Sequencing of both genes revealed a structure characteristic of fungal laccases; the laccase is composed of three cupredoxin domains containing a mononuclear and a trinuclear copper center.

**Figure 7:**
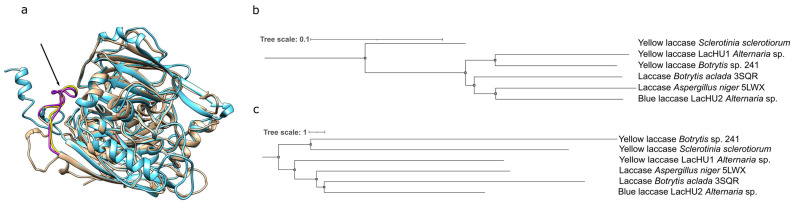
Analysis of LacHU1 sequence and structure. (**a**) LacHU1 and *S. sclerotiorum* yellow laccase (A7EM18) AlfaFold structures comparison. The hydrophobic loop of LacHU1 (yellow) and *S. sclerotiorum* (purple) involved in the binding of a phenolic compound are indicated by arrow. (**b**) Evolution relationship of characterized evolutionarily closest laccases. The Neighbor-Joining algorithm [35] and MEGA7 were used to generate the tree. (**c**) Comparative analysis of protein AlfaFold structures on DALI server [54]. Both trees (**b**,**c**) were visualized with iTOL (https://itol.embl.de/ (accessed on 23 May 2024)).

LacHU1 laccase exhibits unusual spectral properties, lacking the characteristic peak at 600 nm. Yellow laccases, which also lack this absorption peak, are synthesized by the related ascomycetes *Sclerotinia sclerotiorum* [47] and *Botrytis cinerea* [31]. It is hypothesized that yellow laccases are typical blue laccases with a bound phenolic compound acting as a mediator. This hypothesis was proposed by Leontievsky and co-authors in 1997 [48] and was subsequently confirmed by Mot et al., who identified a low-molecular-weight phenolic compound that covalently binds to the flexible amino acid loop near the active site, causing this unusual spectrum (Figure 7) [47]. A homologous amino acid loop exists in the LacHU1 laccase of *Alternaria* sp. HU. Due its spectral properties, LacHU1 could also be characterized as a ‘yellow’ laccase. The phenolic compound is thought to act as a mediator when bound to laccase, reducing the redox potential required to oxidize large substrates. Such a low-molecular-weight compound with a high redox potential (greater than 900 mV) is first oxidized in the active center of the enzyme. The mediator then oxidizes any substrates, e.g., polymers that are unable to interact with the enzyme’s active site due to their size and steric hindrance [26]. The presence of such a mediator broadens the spectrum of oxidizable compounds. A covalently bound mediator further extends the applicability of laccases. As fungi synthesize several different laccases, more and more of these unusual yellow laccases are being discovered [25,47,49,50,51,52], including one of bacterial origin [53]. Interestingly, among the laccases that have been characterized, the yellow laccase LacHU1 is closer in sequence to another yellow laccase from *Botrytis* sp. 241 (Figure 7b), even though these laccases are structurally more distant from each other (Figure 7c). This may indicate that during evolution, determinants for the spectral properties of laccases have evolved, but these changes are not reflected in the active center and spatial structure of the protein.

Another interesting feature to modulate and promote laccase synthesis is its inducibility. Protein synthesis can be increased tens or even hundreds of times by finding the right inducer. Numerous aromatic compounds, including aniline derivates, induce laccase production in fungi [55,56]. For example, 2,5-dimethylaniline increased laccase production in *Pycnoporus cinnabarinus, Pleurotus sajor-casu* [57,58] as well as synthesis of LacHU2 laccase in *Alternaria* sp. Moreover, while screening a number of anilines derivates, we found that 3,4-dimethylaniline most strongly induced the synthesis of LacHU2. The laccase-mediated transformation of 2,5-dimethylaniline or other lignin degradation products leads to the formation of numerous oligomers through oxidative coupling, thus reducing their oxidizing toxicity. This indicates that LacHU2 laccase could be involved in the protection against the toxic effects of anilines.

Laccases are usually active towards a broad spectrum of substrates. The catalytic efficiency of *Alternaria sp.* HU laccases was moderate, as is typical for asco-laccases, but individually, *Alternaria* sp. HU laccases oxidized both natural and synthetic non-phenolic and phenolic substrates effectively. These enzymes efficiently oxidized quercetin, myricetin and fisetin. These flavonoids are naturally found in cotton and need to be removed in the bleaching process. The oxidation of flavonoids by laccase enzymes could be an alternative to the commonly used hydrogen peroxide, which damages cotton fibers [59,60,61]. Thus, LacHU1 and LacHU2 have potential applicability in the bleaching industry. For better adaptation to catalysis, we attempted to immobilize LacHU1 and LacHU2 on magnetic nanoparticles by the adsorption method, which usually does not modify the natural conformation of an enzyme [62]. Despite the loss in efficiencies and gradually decreased activity of the laccases, the immobilization allowed the enzymes to be used multiple times. Hence, after 10 cycles of reuse, the immobilized LacHU1 and LacHU2 retained 18% and 38% of their activities, respectively. Compared to the nanoparticle-immobilized laccases described previously, the reusability of LacHU1 and LacHU2 is similar or greater, as shown in Table S1. The pH optimum of the immobilized laccases, LacHU1 and LacHU2, shifted to a more alkaline region. The same results were obtained with the non-blue laccase from *Methylobacterium extorquens* (Melac13220) on Fe_3_O_4_ nanoparticles using an aldehyde tag [44]. Additionally, a shift in the optimum pH following laccase immobilization has been reported in previous studies [63,64]. This shift can be attributed to the ionic interaction between the laccase and the charged surface of the magnetic nanoparticles [65].

## 5. Conclusions

Under the studied conditions, *Alternaria* sp. HU produced two laccases, LacHU1 (yellow laccase) and LacHU2 (blue laccase). Different growth conditions induced these laccases. Both enzymes had broad substrate specificity and similar kinetic parameters, though LacHU1 was more catalytically efficient. LacHU2 tolerated high salt concentrations and was more suitable for immobilization on magnetic nanoparticles. *Alternaria* sp. laccases could be used to oxidize flavonoids or in the treatment of water with a high concentration of salts.

## Figures and Tables

**Table 1:** Purification of laccases LacHU1 and LacHU2.

Purification Step	Volume, mL	Total Activity, u	Total Protein, mg	Specific Activity, U/mg	Purification Factor, Fold	Yield, %
**LacHU1**						
**Culture liquid**	4000	2210	3200	0.7	1	100
**Ultrafiltration**	262	1540	330	4.7	7	69.6
**PHE FF**	4	1160	61	19	27	52.5
**Source 15Q**	15	960	10.5	91	132	43.4
**Source15PHE**	1	566	4.0	142	205	25.6
**LacHU2**						
**Culture liquid**	960	7104	701	10	1	100
**Ultrafiltration**	248	6795	221	31	3	96
**PHE FF**	10	5403	12.4	436	43	76
**Source 15Q**	0.7	5247	3.2	1630	160	74

**Table 2:** Kinetic parameters of purified *Alternaria* sp. HU laccase isoenzymes.

Substrate	Kinetic Parameters					
	K_M,_ µM		k_cat_, S^−1^		k_cat_/K_M_, M^−1^·s^−1^	
	LacHU1	LacHU2	LacHU1	LacHU2	LacHU1	LacHU2
Non-phenolic compounds						
ABTS	60.0 ± 10.0	(5.6 ± 0.6) × 10^3^	88.0 ± 5.0	440.0 ± 43.0	(1.4 ± 0.1) × 10^6^	(8.0 ± 1.0) × 10^4^
*N,N’*-Dimethylamine-4-(4-morpholine)benzene	90.0 ± 10.0	(1.0 ± 0.2) × 10^4^	100.0 ± 4.0	130.0 ± 20.0	(1.1 ± 0.1) × 10^6^	(1.3 ± 0.4) × 10^4^
2-(10*H*-phenoxazin-10-yl)ethanol	40.0 ± 10.0	200.0 ± 50.0	34.0 ± 3.0	55.0 ± 3.0	(9.0 ± 0.1) × 10^5^	(3.0 ± 1.0) × 10^5^
*p*-Phenylenediamine	340.0 ± 40.0	(3.6 ± 0.8) × 10^3^	30.0 ± 1.0	5.4 ± 0.3	(9.0 ± 0.1) × 10^4^	(1.5 ± 0.4) × 10^3^
Promazine hydrochloride	(3.8 ± 0.5) × 10^3^	(2.6 ± 0.6) × 10^3^	10.0 ± 3.0	3.3 ± 0.1	(3.0 ± 1.0) × 10^3^	(1.3 ± 0.3) × 10^3^
Potassium hexacyanoferrate (II)	nd ^1^	(5.9 ± 1.7) × 10^5^	nd	152.0 ± 21.0	(1.7 ± 0.1) × 10^3^	(3.0 ± 1.0) × 10^2^
Phenolic compounds						
Syringaldazine	1.0 ± 0.1	13.0 ± 3.0	36.0 ± 1.0	5.8 ± 0.7	(5.7 ± 0.5) × 10^7^	(4.5 ± 1.0) × 10^4^
Quercetin	2.0 ± 0.4	80.0 ± 3.0	24.0 ± 1.0	500.0 ± 140.0	(1.3 ± 0.2) × 10^7^	(1.5 ± 0.1) × 10^5^
Myricetin	2.0 ± 0.3	15.0 ± 3.0	28.0 ± 5.0	200.0 ± 0.07	(1.3 ± 0.2) × 10^7^	1.2 ± 0.04) × 10^4^
2,6-DMP	10.0 ± 2.0	(4.3 ± 0.3) × 10^3^	33.0 ± 1.0	204.0 ± 6.0	(3.4 ± 0.4) × 10^6^	(5.0 ± 1.0) × 10^4^
Fisetin	15.0 ± 1.0	27.0 ± 2.0	21.0 ± 2.0	128.0 ± 13.0	(1.4 ± 0.1) × 10^6^	(5.5 ± 0.3) × 10^4^
1-naphthol	100.0 ± 10.0	nd	130.0 ± 9.0	nd	(1.2 ± 0.1) × 10^6^	(1.0 ± 0.1) × 10^2^
Caffeic acid	50.0 ± 3.0	400.0 ± 30.0	27.0 ± 1.0	161.0 ± 33.0	(6.0 ± 0.1) × 10^5^	(4.0 ± 1.0) × 10^5^
Ferulic acid	50.0 ± 4.0	230.0 ± 30.0	28.0 ± 1.5	9.0 ± 1.0	(6.0 ± 0.1) × 10^5^	(4.3 ± 1.0) × 10^4^
Syringic acid	40.0 ± 0.5	nox ^2^	20.0 ± 1.0	nox	(5.0 ± 0.1) × 10^5^	nox
Kaempferol	110.0 ± 22.0	120.0 ± 10.0	38.0 ± 14.0	26.0 ± 4.0	(3.0 ± 1.0) × 10^5^	(7.0 ± 1.0) × 10^4^
Gallic acid	nd	nox	nd	nox	(3.0 ± 0.1) × 10^5^	nox
Chlorogenic acid	100.0 ± 1.0	90.0 ± 40.0	13.5 ± 0.2	38.0 ± 12.0	(1.3 ± 0.1) × 10^5^	(4.3 ± 1.0) × 10^5^
Hydroquinone	540.0 ± 10.0	(1.3 ± 0.2) × 10^4^	62.0 ± 1.0	400.0 ± 46.0	(1.1 ± 0.3) × 10^5^	(3.0 ± 1.0) × 10^4^
Catechol	(1.8 ± 0.2) × 10^3^	(2.0 ± 0.4) × 10^4^	105.0 ± 5.0	900.0 ± 160.0	(6.0 ± 0.1) × 10^4^	(4.4 ± 1.0) × 10^4^

^1^ nd—not determined. K_M_ and V_max_ constants cannot be determined because a linear dependence on the substrate concentration was obtained. This makes it possible to calculate only one parameter (k_cat_/K_M_ =Vo /[S] [E] = slope/[E]). ^2^ nox—not oxidized. Laccase did not oxidize a substrate.

**Table 3:** Kinetic constants of free and immobilized LacHU1 and LacHU2 isoenzymes on ABTS.

Laccase	K_M,_ μM	k_cat,_ s^−1^	k_cat_/K_M,_ M^−1^S^−1^
Free LacHU1	60 ± 10	88.0 ± 5.0	(1.4 ± 0.1) × 10^6^
Immobilized LacHU1	65 ± 9.0	3.0 ± 0.14	(4.6 ± 1.0) × 10^4^
Free LacHU2	(5.6 ± 0.6) × 10^3^	440.0 ± 43.0	(8.0 ± 1.0) × 10^4^
Immobilized LacHU2	(2.2 ± 0.5) × 10^3^	100.0 ± 15.0	(4.6 ± 3.0) × 10^4^

## Data Availability

The original contributions presented in the study are included in the article/Supplementary Materials, further inquiries can be directed to the corresponding author.

## Supplementary Materials

The following supporting information can be downloaded at: https://www.mdpi.com/article/10.3390/jof10080559/s1, Figure S1: Analysis of the kinetic parameters of the purified *Alternaria* sp. HU laccase isoenzymes (N = 3); Figure S2: Effect of pH on activity of LacHU1 () and LacHU2 (). (a) syringaldazine; (b) 1-naphthol; (c) ferulic acid and (d) myricetin as substrates in 60 mM Britton-Robinson buffer, 25 °C. The highest activity was equated to 100%.; Figure S3: Effect of pH on stability of LacHU1 () and LacHU2 (). Laccases were incubated for 20 hours in 60 mM Britton-Robinson buffer, at 25 °C, ABTS was used as a substrate. The highest activity was equated to 100%.; Figure S4: Dependence of enzymatic activity on laccase concentration used for immobilization.; Table S1: Comparison of the retained activity of laccase immobilized on different matrices after 10 cycles of reuse.
