# Supplementary material for: Blue and Yellow Laccases from Alternaria sp. Strain HU: Characterization and Immobilization on Magnetic Nanoparticles
Source: J Fungi (Basel). 2024 Aug 8;10(8):559. doi: 10.3390/jof10080559 (PMC11355796; doi:10.3390/jof10080559)
Supplement: Supplementary file 1 [file jof-10-00559-s001.zip › jof-3105595-supplementary.pdf]

Supplementary material

Blue and Yellow Laccases from *Alternaria* sp. Strain HU: Characterization and Immobilization on Magnetic Nanoparticles

Ingrida Radveikienė *, Regina Vidžiūnaitė, Rolandas Meškys and Vida Časaitė

Life Sciences Center, Institute of Biochemistry, Vilnius University, Sauletekio Av. 7, 10257 Vilnius, Lithuania
* Correspondence: i.radveikiene@gmail.com

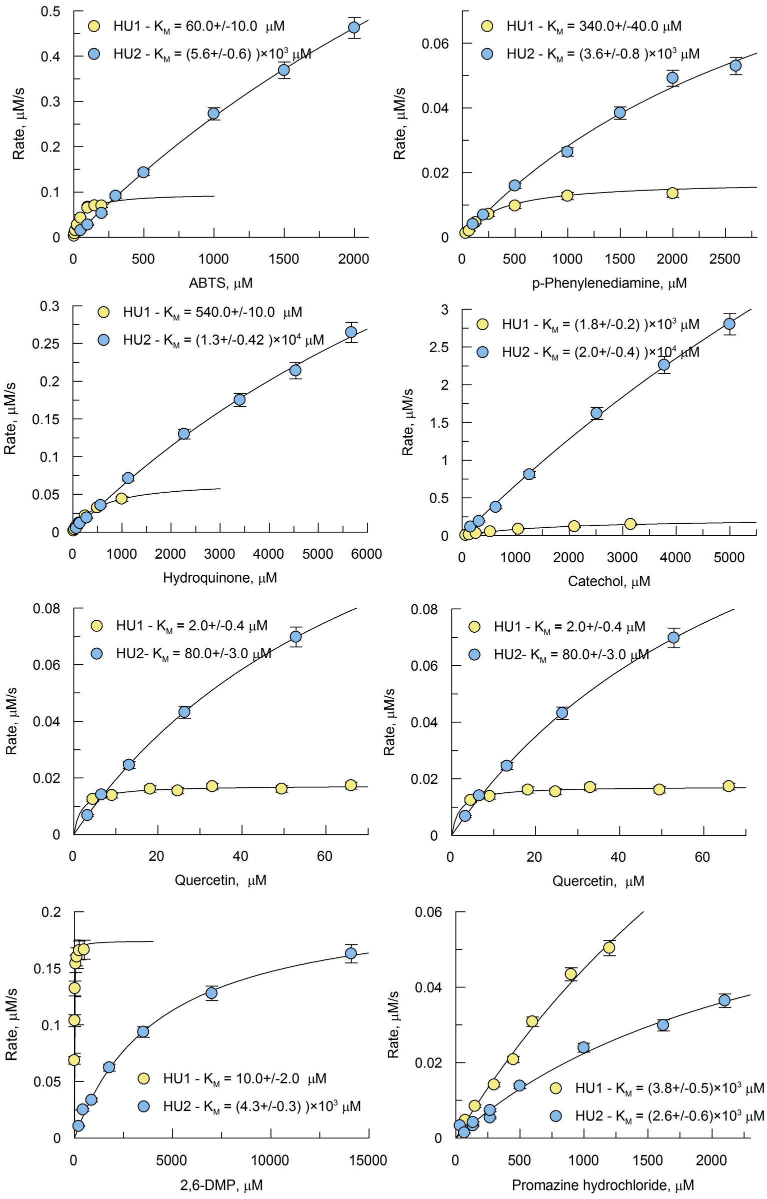

Figure S1. Analysis of the kinetic parameters of the purified *Alternaria* sp. HU laccase isoenzymes (N=3).

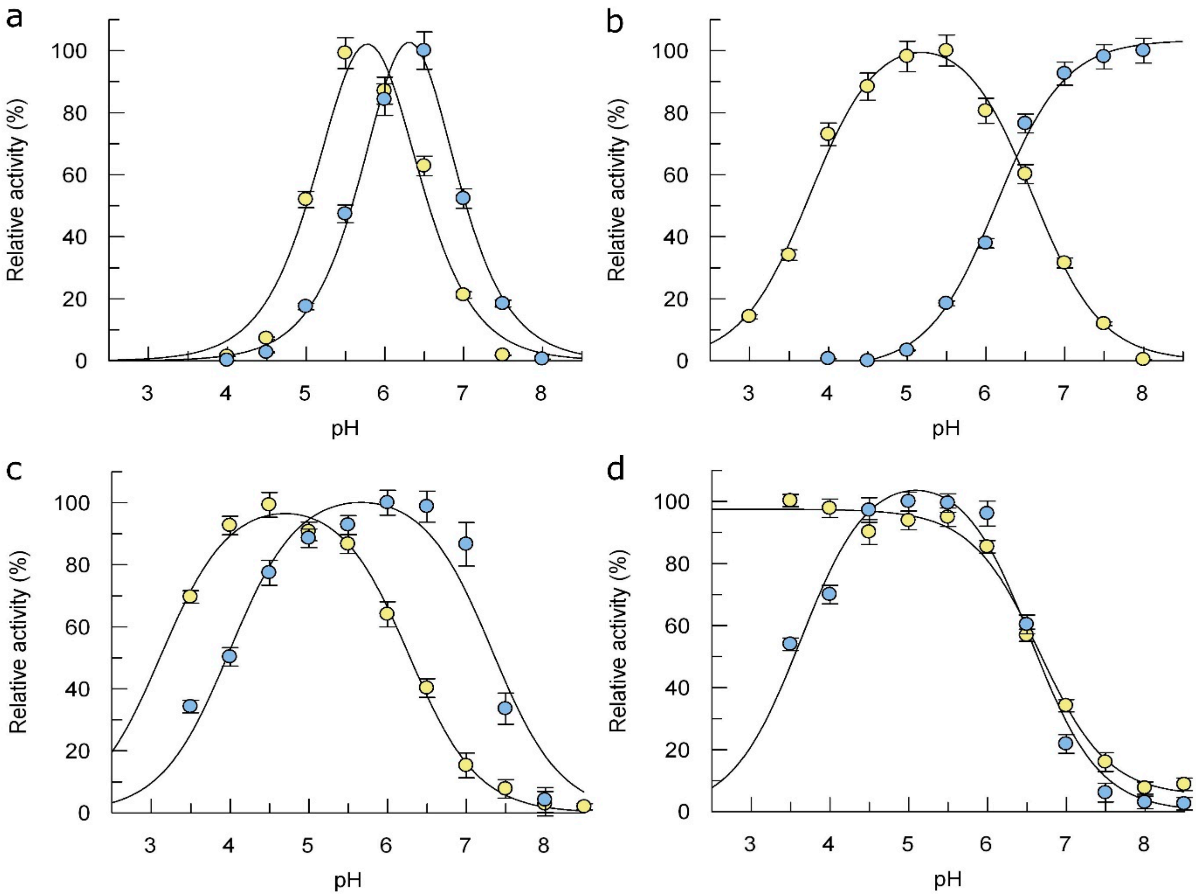

Figure S2. Effect of pH on activity of LacHU1 ( ) and LacHU2 ( ). (a) syringaldazine; (b) 1-naphthol; (c) ferulic acid and (d) myricetin as substrates in 60 mM Britton-Robinson buffer, 25 °C. The highest activity was equated to 100%.

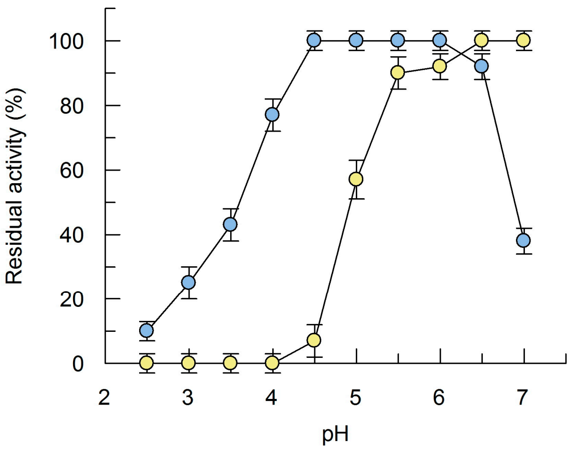

Figure S3. Effect of pH on stability of LacHU1 ( ) and LacHU2 ( ). Laccases were incubated for 20 hours in 60 mM Britton-Robinson buffer, at 25 °C, ABTS was used as a substrate. The highest activity was equated to 100%.

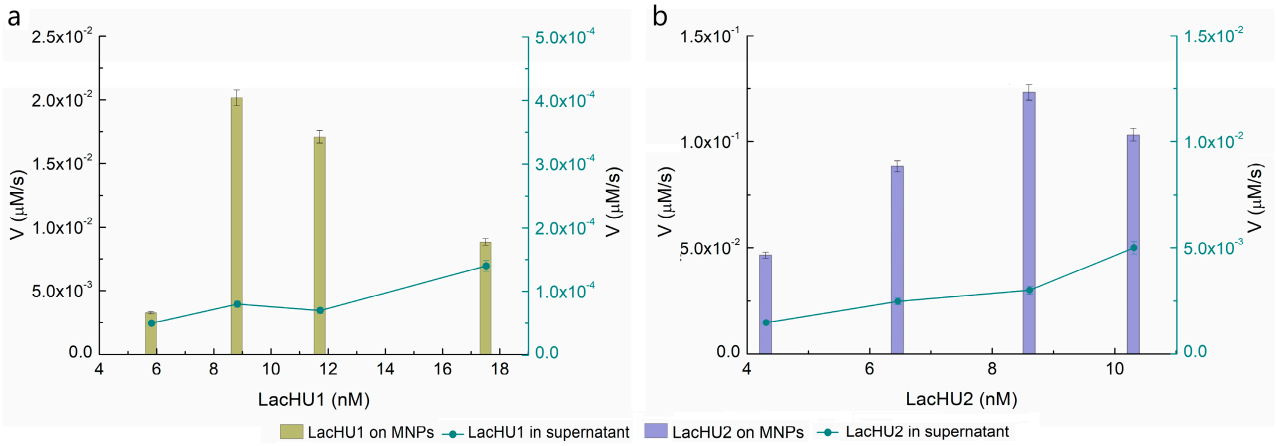

Figure S4. Dependence of enzymatic activity on laccase concentration used for immobilization.

Table S1. Comparison of the retained activity of laccase immobilised on different matrices after 10 cycles of reuse.

Enzyme	Matrix	Method	Retained activity after 10 cycles, %	References
Rhus vernicifera laccase	Fe_3O_4	Adsorption	21.3	[64]
Rhus vernicifera laccase	$Fe_3O_4@MoS_2$	Adsorption	62.0	[65]
Rhus vernicifera laccase	Fe_2O_3	Covalent	82.9	[66]
Trametes versicolor laccase	Amino-functionalized Fe_3O_4	Covalent	32.0	[67]
Methylobacterium extorquens laccase	Fe_2O_3	Covalent	81.1	[42]
LacHU1	Fe_3O_4	Adsorption	18.0	This study
LacHU2	Fe_3O_4	Adsorption	38.0	This study
